# Supplementary material for: O2A: One-Shot Observational Learning with Action Vectors
Source: Front Robot AI. 2021 Aug 2;8:686368. doi: 10.3389/frobt.2021.686368 (PMC8367442; doi:10.3389/frobt.2021.686368)
Supplement: Supplementary file 1 [file DataSheet1.pdf]

# Supplementary Material

## 1 ACTION VECTOR EXTRACTOR: MODEL ARCHITECTURE AND PRE-TRAINING DETAILS

The architecture of the 3D-CNN model used is given in Table S1. Variable NC denotes the number of classes. The details of pre-training the model for action classification are given in Table S2.

**Table S1.** 3D-CNN model used for action vector extraction used in our experiments. 'NC' denotes the number of classes

| Layer | Type | Kernel size | Input size | Output size |
|---|---|---|---|---|
| | | | | |
| conv1 | Conv3D | (3, 3, 3) | (16, 112, 112, 3) | (16, 112, 112, 64) |
| pool1 | MaxPooling3D | (1, 2, 2) | (16, 112, 112, 64) | (16, 56, 56, 64) |
| | | | | |
| conv2 | Conv3D | (3, 3, 3) | (16, 56, 56, 64) | (16, 56, 56, 128) |
| pool2 | MaxPooling3D | (2, 2, 2) | (16, 56, 56, 128) | (8, 28, 28, 128) |
| | | | | |
| conv3a | Conv3D | (3, 3, 3) | (8, 28, 28, 128) | (8, 28, 28, 256) |
| conv3b | Conv3D | (3, 3, 3) | (8, 28, 28, 256) | (8, 28, 28, 256) |
| pool3 | MaxPooling3D | (2, 2, 2) | (8, 28, 28, 256) | (4, 14, 14, 256) |
| | | | | |
| conv4a | Conv3D | (3, 3, 3) | (4, 14, 14, 256) | (4, 14, 14, 512) |
| conv4b | Conv3D | (3, 3, 3) | (4, 14, 14, 512) | (4, 14, 14, 512) |
| pool4 | MaxPooling3D | (2, 2, 2) | (4, 14, 14, 512) | (2, 7, 7, 512) |
| | | | | |
| conv5a | Conv3D | (3, 3, 3) | (2, 7, 7, 512) | (2, 7, 7, 512) |
| conv5b | Conv3D | (3, 3, 3) | (2, 7, 7, 512) | (2, 7, 7, 512) |
| zeropad5 | ZeroPadding3D | (0, 1, 1) | (2, 7, 7, 512) | (2, 8, 8, 512) |
| pool5 | MaxPooling3D | (2, 2, 2) | (2, 8, 8, 512) | (1, 4, 4, 512) |
| flatten1 | Flatten | - | (1, 4, 4, 512) | (8192) |
| | | | | |
| fc6 | Dense | (4096) | (8192) | (4096) |
| fc7 | Dense | (4096) | (4096) | (4096) |
| fc8 | Dense | (NC) | (4096) | (NC) |

**Table S2.** Pre-training details for the NN:UCF101 model

| Parameter | Value |
|---|---|
| **Number of classes (NC)** | 101 |
| **Batch size** | 16 |
| **Input size** | (16, 16, 112, 112, 3) |
| **Output size** | (16, 101) |
| **GPUs used** | 2 x Nvidia K80 |
| **Training time** | 48 hrs |
| **Optimizer** | ADAGRAD Duchi et al. (2011) |
| **Learning rate** | 0.001 |
| **Number of training examples** | 9,990 |
| **Number of validating examples** | 3,330 |
| **Total number of trainable parameters** | 78,409,573 |
| **Number of epochs** | 119 |
| **Best validation accuracy** | 60.72% |

## 2 DDPG ALGORITHM

Details of the DDPG algorithm used in the simulation experiment are given here. We use architectures similar to Lillicrap et al. (2015) for the actor and critic networks. The hyper-parameters used are given in Table S3.

**Table S3.** DDPG hyperparameters used

| Hyperparameter | Value |
|---|---|
| **Actor learning rate** | 0.0001 |
| **Critic learning rate** | 0.001 |
| **State size** | 4608 |
| **Action size** | 3 |
| **Optimiser used** | ADAM |
| **Gamma ($\gamma$)** | 0.99 |
| **Tau ($\tau$)** | 0.001 |
| **Mini batch size** | 64 |
| **Replay buffer size** | 10,000 |

## 3 OPTIMAL NUMBER OF CLUSTERS

Here we find the the optimal number of clusters (optimal K value) and the corresponding optimal ARI score for clustering analysis. We calculate the ARI scores by performing clustering analysis for each value of K from 1 to the total number samples in the dataset (51 for LMD). The results are plotted in figure S1. The optimal K value is selected corresponding to the highest ARI score and in case of multiple peaks, the K value closer to the number of classes in the dataset (3 for LMD) is selected. The optimal ARI score for each layer is compared in Table S4. The results are in agreement with the other experiments and show that the features from pool5 and fc6 layers of the NN:UCF101 model are best suited to be used as the action vector.

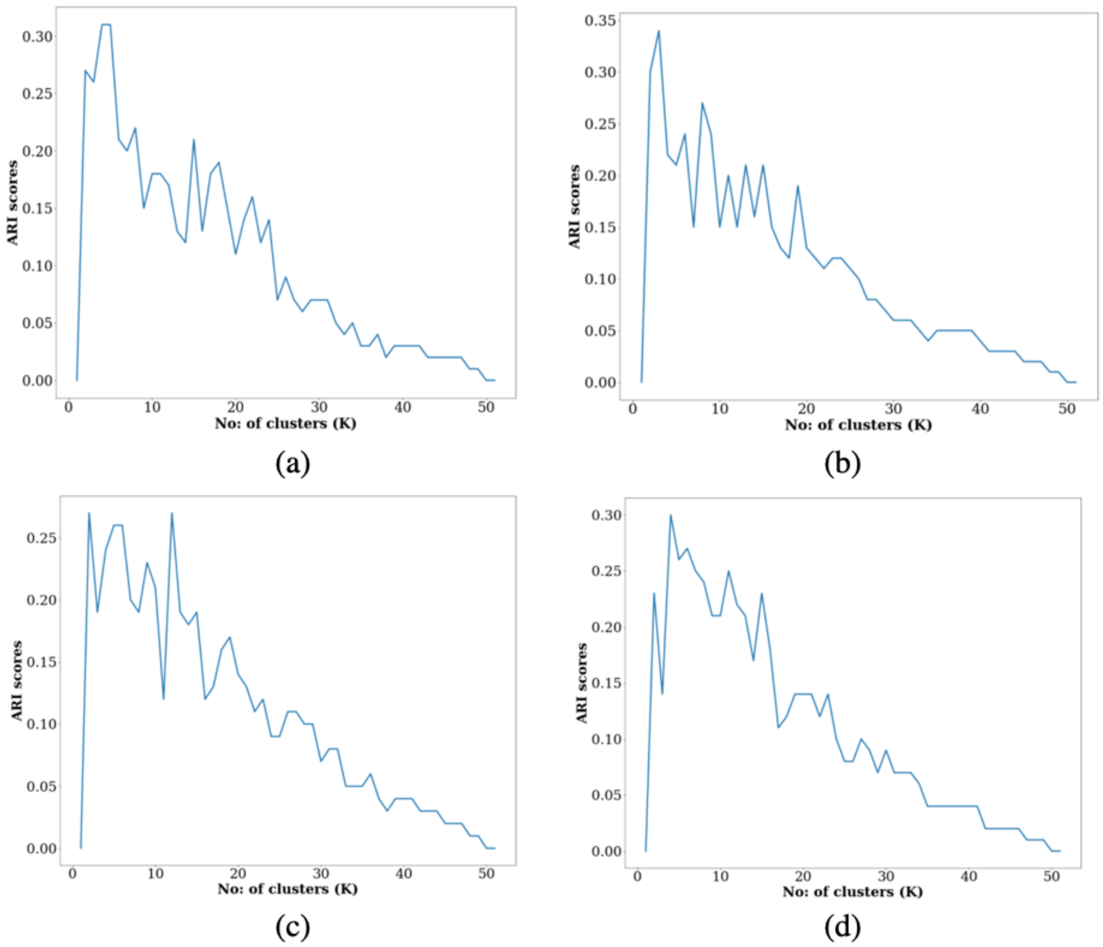


**Figure S1.** ARI scores after clustering analysis for different K values (1 to 51) when features from (a) pool5 (b) fc6 (c) fc7 and (d) fc8 layers of NN:UCF101 are used as the action vector.

**Table S4.** Optimal ARI scores corresponding to optimal K values. Results show that the features from layers pool5 and fc6 of the NN:UCF101 model are best suited to be used as action vectors.

| Layer | Optimal K value | Optimal ARI score |
|---|---|---|
| **pool5** | 4 | **0.31** |
| **fc6** | 3 | **0.34** |
| **fc7** | 2 | 0.27 |
| **fc8** | 4 | 0.30 |

## 4 TRAJECTORY MAPS

Trajectory maps obtained during reinforcement learning of the task of pushing, when $O_2A$ action vector extractor is pre-trained with UCF101 dataset (NN:UCF101 (pool5, fc6)) and with MIME dataset (NN:MIME (pool5, fc6)) are shown in Figure S2.

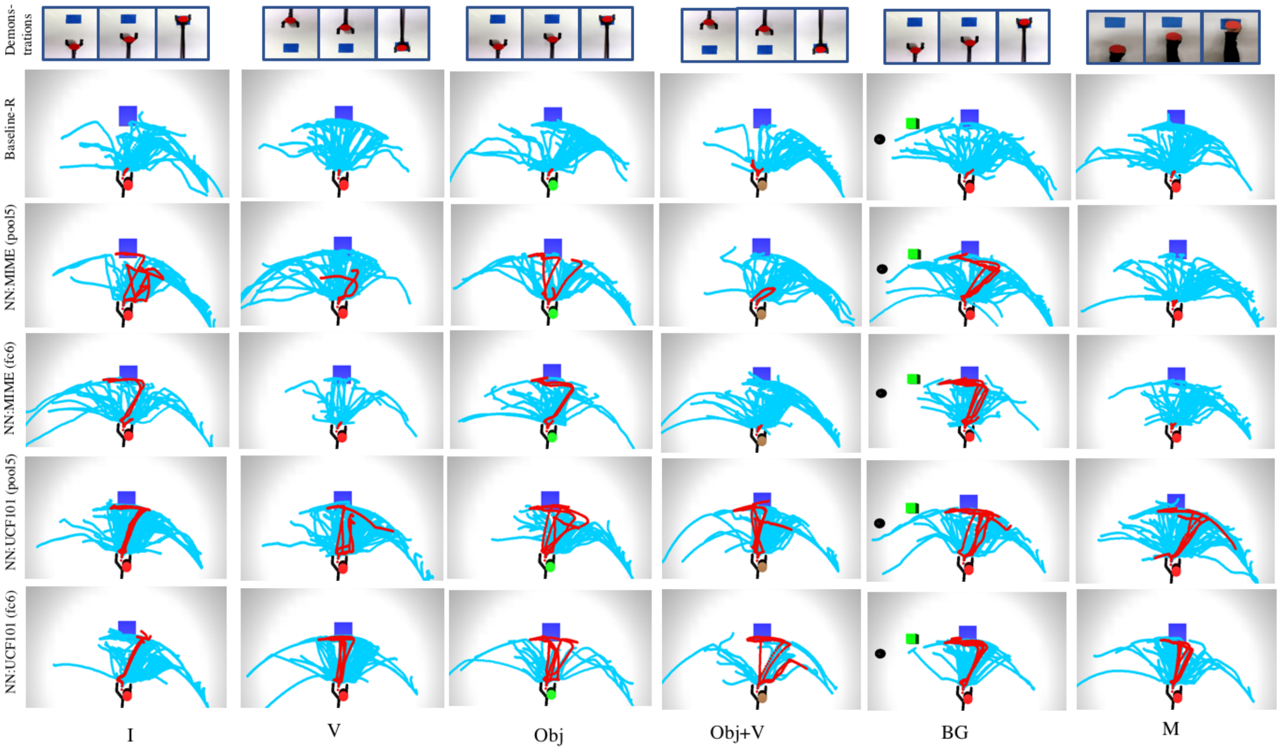


**Figure S2.** Trajectory maps obtained during reinforcement learning of the task of pushing, when $O_2A$ action vector extractors are pre-trained with UCF101 dataset (NN:UCF101 (pool5, fc6)) and with MIME dataset (NN:MIME (pool5, fc6)). NN:UCF101 provides high rewards for desired trajectories for all the domain shifts (I, V, Obj, Obj+V, BG, M). However, NN:MIME performs poorly when viewpoint of observation (V, Obj+V) and morphology of the manipulator (M) changes.

## 5 BACKGROUND COLOUR

We plotted trajectory maps for $O_2A$ with domain shifts characterised by changes in background colour. The results are given in Figures S3 and S4. BG-A, BG-B denotes domain shifts characterised by changes in background colour, while BG-A+V denote changes in background colour and viewpoint. High rewards were obtained for desired trajectories for the tasks of reaching and pushing for all the cases.

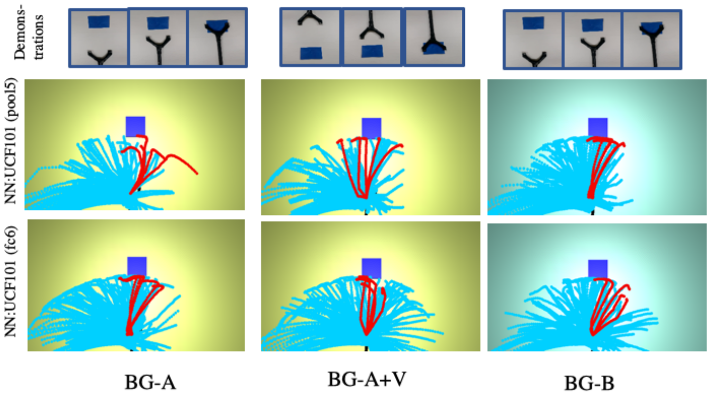


**Figure S3.** Trajectory maps for $O_2A$ obtained for the task of reaching for domain shifts characterised by changes in background color (BG-A, BG-B) and viewpoint (BG-A+V). High rewards are obtained for desired trajectories for all the cases.

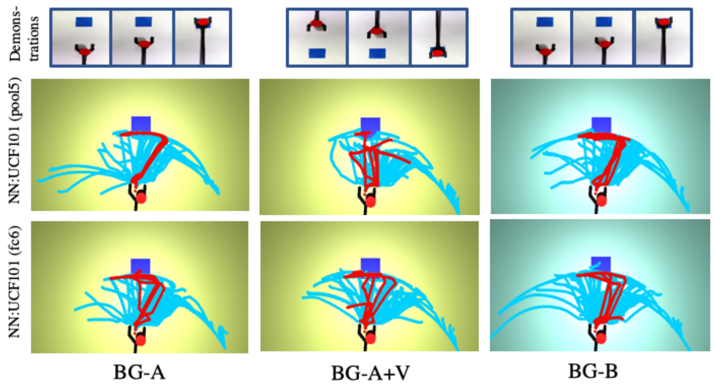


**Figure S4.** Trajectory maps for $O_2A$ obtained for the task of pushing for domain shifts characterised by changes in background color (BG-A, BG-B) and viewpoint (BG-A+V). High rewards are obtained for desired trajectories for all the cases.

## 6 REACH - PUSH

We conducted the experiment with the reach-push task to show that $O_2A$ can generate high rewards for desired trajectories for more complex tasks beyond reaching and pushing. In reach-push task, the object is far from the manipulator and not aligned with the target. The manipulator has to first reach the object (reach phase) and then push it to the target (push phase). We manually collected a set of video samples showing varying degrees of task completion and calculated reward values with respect to a task demonstration. Figure S5 shows snapshots of the video samples including the task demonstration used. Reward values are calculated for each pair of videos (D-A to D-E) and is plotted in Figure S6. The reward value increases as

the task moves towards completion. This shows that $O_2A$ reward function can successfully model more complex tasks beyond reaching and pushing.

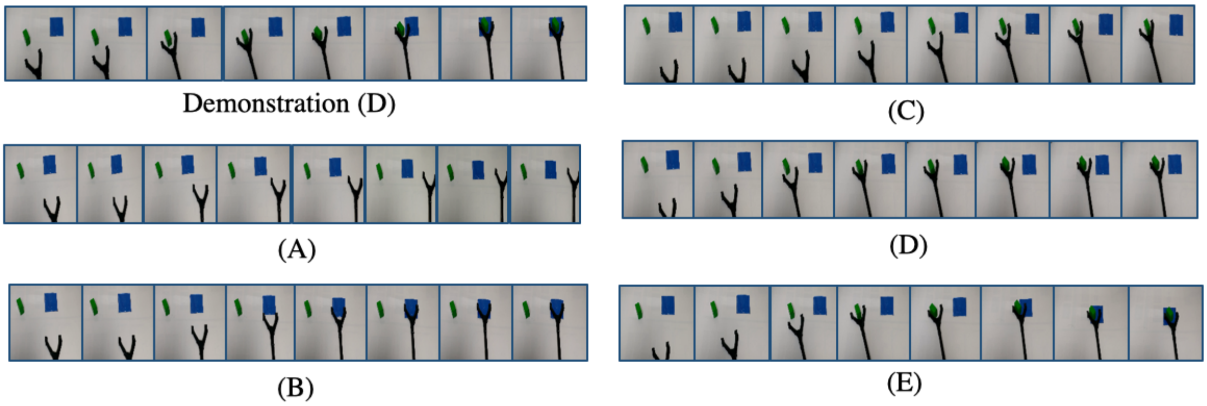


**Figure S5.** Snapshots of the video samples of the reach-push task collected. It includes a demonstration (D) and video samples showing varying degrees of task completion (A-E).

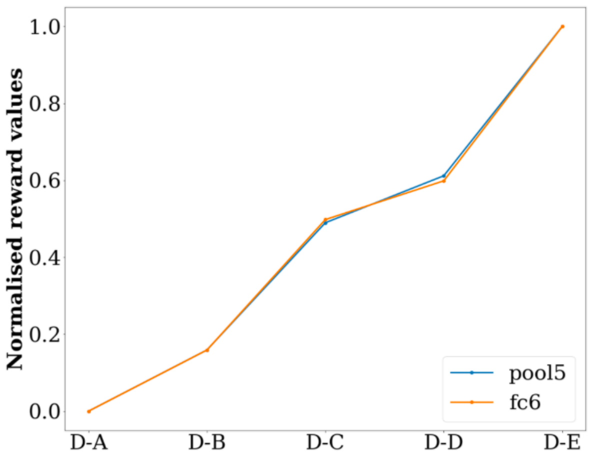


**Figure S6.** Normalised reward values for video pairs D-A to D-E. Higher rewards are obtained when task moves towards completion, showing $O_2A$ can successfully model the more complete reach-push task as well.

## 7 STO ALGORITHM

Algorithm 1 gives a detailed step by step implementation of STO.

**Algorithm 1:** Stochastic Trajectory Optimization

**Input** : $\mathbf{U_0}$: Initial control sequence containing $n$ steps
**Input** : $\mathbf{U}$: Candidate control sequence containing $n$ steps
**Parameters** : $K$: Number of noisy trajectory rollouts
$\mathcal{N}(\mathbf{0}, \nu)$: Gaussian noise with variance $\nu$
$C_{thresh}$: Success definition in terms of cost
$I_{max}$: Maximum number of iterations

```
U ← U_0
C ← TrajectoryRollout(U)
while I_max not reached and C > C_thresh do
    for k ← 0 to K − 1 do
        δU^k ← N(0, ν)                      ▷ Random control sequence variation
        U^k = U + δU^k
        C^k ← TrajectoryRollout(U^k)
    U, C ← MinCostTraj(U, {U^0, ..., U^{K−1}}, C, {C^0, ... C^{K−1}})
return U
```

## 8 WEBSITE DETAILS

Project website: page-main
Results: page-results

## REFERENCES


Duchi, J., Hazan, E., and Singer, Y. (2011). Adaptive subgradient methods for online learning and stochastic optimization. *Journal of Machine Learning Research* 12, 2121–2159

Lillicrap, T. P., Hunt, J. J., Pritzel, A., Heess, N., Erez, T., Tassa, Y., et al. (2015). Continuous control with deep reinforcement learning. In *International Conference on Learning Representations*
